# Impact of Chile’s Explicit Health Guarantees Policy on breast cancer mortality: an interrupted time series analysis (1990–2024)

**DOI:** 10.3389/fonc.2026.1801070

**Published:** 2026-10-13

**Authors:** Matilde Maddaleno Herrera, Christian García-Calavaro, Felipe Elorrieta, Angélica Verdugo Sobral, Ignacio Toledo-Ordóñez

**Affiliations:** 1 Public Health Center Program, Faculty of Medical Sciences, University of Santiago of Chile, Santiago, Chile; 2 Departamento de Planificación y Control de Gestión, Servicio de Salud Metropolitano Oriente, Santiago, Chile; 3 Department of Mathematics and Computer Science, Faculty of Sciences, University of Santiago of Chile, Santiago, Chile; 4 Health Division, Corporación de Educación, Salud y Atención de Menores de Conchalí (CORESAM), Santiago, Chile

**Keywords:** breast cancer, breast neoplasms, Explicit Health Guarantees, health policy evaluation, interrupted time series

## Abstract

**Background:**

Breast cancer mortality in Chile has shown divergent trends over recent decades, with declining age-adjusted rates alongside increasing crude mortality driven by population aging. In 2005, Chile incorporated breast cancer into the Explicit Health Guarantees Regime (AUGE/GES), ensuring universal access, timeliness, quality standards, and financial protection across the continuum of care. Whether this policy produced an independent, detectable impact on population-level breast cancer mortality remains uncertain.

**Methods:**

We conducted an interrupted time series (ITS) analysis of female breast cancer mortality in Chile from 1990 to 2024. Annual death counts were obtained from national vital statistics and modeled using negative binomial regression with population offsets. Age-standardized mortality rates were calculated using the 2017 Chilean population as the reference. ITS models estimated changes in level and slope associated with two intervention points: the consolidation of the National Breast Cancer Program in 1995 and the implementation of AUGE/GES in 2005. Analyses were performed for the total female population and by age group.

**Results:**

While the absolute number of deaths and crude mortality rates increased over time, age-standardized breast cancer mortality declined steadily throughout the study period. A significant change in trend was observed after 1995, consistent with the early programmatic organization of breast cancer services. In contrast, no additional statistically significant change in level or slope was detected after 2005 at the population level or among women aged ≥40/≥50 years. Age-stratified analyses revealed a more pronounced post-2005 decline among women aged 65–69 and ≥80 years, suggesting age- and cohort-specific effects.

**Conclusions:**

Breast cancer mortality risk in Chile has declined primarily due to long-term secular trends and early programmatic strengthening, while population aging has driven increasing crude mortality. The AUGE/GES reform has consolidated access and financial protection, with heterogeneous benefits in older cohorts, but without generating a detectable additional inflection in national mortality trends. Further mortality reductions will require integrated, equity-oriented strategies emphasizing organized screening, timely diagnosis, and high-quality treatment across the life course.

## Introduction

Breast cancer is the most common malignancy and the leading cause of cancer-related death among adult women in Chile. In 2022, age-standardized incidence and mortality rates reached 38.2 and 10.3 per 100,000 women, respectively (1). Over recent decades, the country has experienced a sustained increase in incidence alongside stabilization or modest declines in age-adjusted mortality. This pattern, consistent with trends observed in many middle- and high-income countries, has been attributed to the expansion of early detection, advances in oncologic treatments, and the progressive strengthening of specialized cancer care networks (2–5). However, these improvements have coexisted with persistent socioeconomic and territorial inequalities in incidence, mortality, and survival, driven by disparities in timely access to diagnosis and treatment (6–8).

Chile’s organized public health response was consolidated in 1995 through the National Breast Cancer Program, which aimed to reduce mortality by promoting early detection and ensuring timely, high-quality treatment. At that time, breast cancer accounted for approximately 932 deaths annually and represented the second leading cause of cancer death among women (9, 10). The program initially targeted women aged 35–64 years who were users of the public health system and combined detection strategies at the primary care level with comprehensive treatment pathways. Its early design relied on clinical breast examination and progressively incorporated mammography as a complementary screening tool (2, 9, 11).

Between the mid-1990s and the early 2000s, Chile experienced notable improvements in diagnostic capacity, mammography coverage, and clinical outcomes. Expansion of public infrastructure and sustained increases in mammography utilization were associated with a shift toward earlier-stage diagnosis, a reduction in advanced cases, and declines in age-adjusted mortality, particularly among age groups prioritized by public policy (10).

A major turning point in Chilean health policy occurred in 2005 with the implementation of the General Regime of Explicit Health Guarantees (AUGE/GES; *Garantías Explícitas en Salud*), a universal coverage reform that transformed the management of prioritized health problems into legally enforceable rights. Structured around guarantees of access, timeliness, financial protection, and quality, AUGE/GES incorporated breast cancer from its inception and covered the full continuum of care, from diagnostic suspicion to post-treatment follow-up (12, 13).

The initial benefits package included specialist consultation, imaging, histopathological confirmation, oncologic surgery, radiotherapy, systemic therapies, and structured follow-up, with defined maximum timeframes for each stage (14, 15). Subsequent updates expanded financial coverage and therapeutic complexity, incorporated regimens differentiated by biological subtype, strengthened mammography-based screening strategies, broadened the target population, and introduced measures to reduce administrative and economic barriers to care (5, 15, 16).

In this context, breast cancer mortality in Chile has shown a sustained downward trend over the past two decades. Nevertheless, mammography coverage remains below that observed in countries of the Organization for Economic Co-operation and Development (OECD), highlighting ongoing challenges in achieving effective, equitable, population-based screening (17). Moreover, the overlapping implementation of multiple health system reforms complicates attribution of observed mortality trends to any single policy intervention.

Despite the leading role of AUGE/GES in structuring oncologic care in Chile, robust evidence isolating its specific contribution to changes in breast cancer mortality—independent of pre-existing secular trends and concurrent health system transformations—remains limited. Therefore, this study estimates the impact of AUGE/GES on breast cancer mortality among women aged 15 years and older in Chile using an interrupted time series (ITS) design (18), providing policy-relevant evidence on the effects and limits of universal health coverage reforms in a middle-income country undergoing rapid demographic transition.

## Materials and methods

### Study design

Population-based interrupted time series analysis of annual breast cancer mortality in Chile from 1990 to 2024.

### Population denominators

Population estimates by year, sex, age group (five-year intervals), and region were derived from official national census sources. Projections based on the 2002 Census were used for 1990–2001, and projections based on the 2017 Census were used for 2002–2024. To ensure continuity and internal coherence across census bases, both series were harmonized using a two-step procedure combining growth chain-linking (19) (anchored at 2002) and ratio-splicing with LOESS smoothing (20). Full methodological details are provided in the **Supplementary Material**.

### Mortality data

Mortality data were obtained from the Department of Health Statistics and Information (DEIS), Ministry of Health of Chile. We included all registered female deaths attributed to breast cancer using ICD-9 (1990-1997) and ICD-10 (1997-2024) from official data (1990 to 2022) and from preliminary death records 2023–2024 (ICD codes in the **Supplementary Material**). Annual mortality rates were calculated per 10,000 women overall and by age group, with direct age standardization using the 2017 Chilean female population as the reference.

### Statistical analysis

We applied interrupted time series models (18, 21) using negative binomial regression (22) to account for overdispersion in mortality counts. As an applied example of ITS in-health settings, we considered previous work using interrupted time-series analysis in admissions data (23). The logarithm of the female population was included as an offset, allowing direct estimation of mortality rates and rate ratios. Models included terms for baseline temporal trend, intervention level change, and post-intervention slope change. We fitted separate segmented regression models for two prespecified policy breakpoints: 1995, corresponding to the consolidation of the National Breast Cancer Program, and 2005, corresponding to the implementation of AUGE/GES. The 1995 analysis was intended to characterize an earlier programmatic change that could have shaped the pre-GES mortality trajectory, whereas the 2005 analysis constituted the primary policy evaluation. To mitigate potential multicollinearity between time-trend components, the time variable (Year t) was centered at the respective intervention year.

The model structure used is as follows:


$${log(Rate}_{t}=log(\frac{E(N_{esp})t}{{Population}_{t}})=\beta_{0}+\beta_{1}{Year}_{t}+\beta_{2}{Interv}_{t}+\beta_{3}{(Year}_{t}*{Interv}_{t})+\varepsilon_{t}$$

Where:

- (Rate t) is the estimated rate of N_esp (Expected number of deaths) per population at time t,
- 0 represents the baseline level of the log-rate at Time = 0 (intercept)
- Year t is the time variable centered at each intervention Year t = t - tintervention(1995, 2005)
- 1 represents the pre-intervention trend (the change in log-rate per Year before the intervention).
- (Interv t) is a binary variable (0 before the intervention, 1 after)
- 2 estimates the immediate change in the level of the log-rate following the intervention (or step change).
- (Year t *Interv t) is the interaction term.
- 3 estimates change in the post-intervention trend relative to the pre-intervention trend 1.
- Ɛ t is the error term of the model

Model parameters were interpreted as changes in log-rates, with exponentiated coefficients representing relative changes in mortality rates. Statistical significance was assessed at α = 0.05. We fitted 9 models: for the whole female population, for the female population >50 and for each quinquennial age group: 50-54, 55-59, 60-64, 65-69, 70-74, 75–79 and 80 +. Models were assessed for autocorrelation using Ljung-Box test and for overdispersion using the Deviance/DF ratio (24).

### Ethics statement

This study used aggregated secondary data and did not involve human participants. Institutional approval: Informe Exento N° 533-2025.

## Results

A total of 43,839 breast cancer deaths were reported in Chile between 1990 and 2024. Women accounted for the vast majority of cases, representing 99.1% of all deaths (n = 43,438), while male breast cancer deaths were rare (401 deaths; 0.9%). Mortality before age 30 was uncommon, accounting for approximately 1% of total deaths.

Among women, breast cancer mortality increased markedly after age 50, with approximately 80% of female deaths occurring in women aged 50 years and older. Mortality peaked in the 80 years and older age group, which accounted for 9,639 deaths (22% of all female deaths). Each five-year age group between 50 and 79 years contributed approximately 10–13% of total female deaths, reflecting a clear age gradient in breast cancer mortality (**Table 1**).

**Table 1:** Breast cancer deaths by age group and sex, Chile 1990-2024.

Age Group	Overall N = 43,839*^1^*	Male N = 401*^1^*	Female N = 43,438*^1^*	p-value*^2^* < 0.001
0-4	1 (<0.1%)	0 (0%)	1 (<0.1%)	
5-9	1 (<0.1%)	1 (0.2%)	0 (0%)	
10-14	0 (0%)	0 (0%)	0 (0%)	
15-19	2 (<0.1%)	0 (0%)	2 (<0.1%)	
20-24	16 (<0.1%)	0 (0%)	16 (<0.1%)	
25-29	164 (0.4%)	2 (0.5%)	162 (0.4%)	
30-34	554 (1.3%)	1 (0.2%)	553 (1.3%)	
35-39	1,255 (2.9%)	6 (1.5%)	1,249 (2.9%)	
40-44	2,235 (5.1%)	7 (1.7%)	2,228 (5.1%)	
45-49	3,353 (7.6%)	12 (3.0%)	3,341 (7.7%)	
50-54	4,092 (9.3%)	15 (3.7%)	4,077 (9.4%)	
55-59	4,524 (10%)	24 (6.0%)	4,500 (10%)	
60-64	5,553 (13%)	43 (11%)	5,510 (13%)	
65-69	3,628 (8.3%)	41 (10%)	3,587 (8.3%)	
70-74	4,434 (10%)	71 (18%)	4,363 (10%)	
75-79	4,388 (10%)	49 (12%)	4,339 (10.0%)	
80+	9,639 (22%)	129 (32%)	9,510 (22%)	

^1^ n (%).

^2^ Pearson’s Chi-squared test with simulated p-value (based on 2000 replicates)

Over the study period, crude mortality rate rose from 1.2 to 1.8 deaths per 10,000 women, reflecting the growing absolute burden of breast cancer mortality in the population. No abrupt changes or discontinuities are observed around either 1995 or 2004 in crude indicators. In contrast, age-standardized breast cancer mortality rates showed a sustained downward trend throughout the study period, from 2.13 to 1.10 per 10,000 over the 34 years, halving the rate observed in 1990. This divergence indicates that the observed increase in crude mortality is primarily attributable to population aging, rather than to an increase in age-specific risk of dying from breast cancer.

The standardized trend shows no reversal over time, supporting the presence of a long-term decline in breast cancer mortality risk at the population level (**Figure 1**).

**Figure 1:**
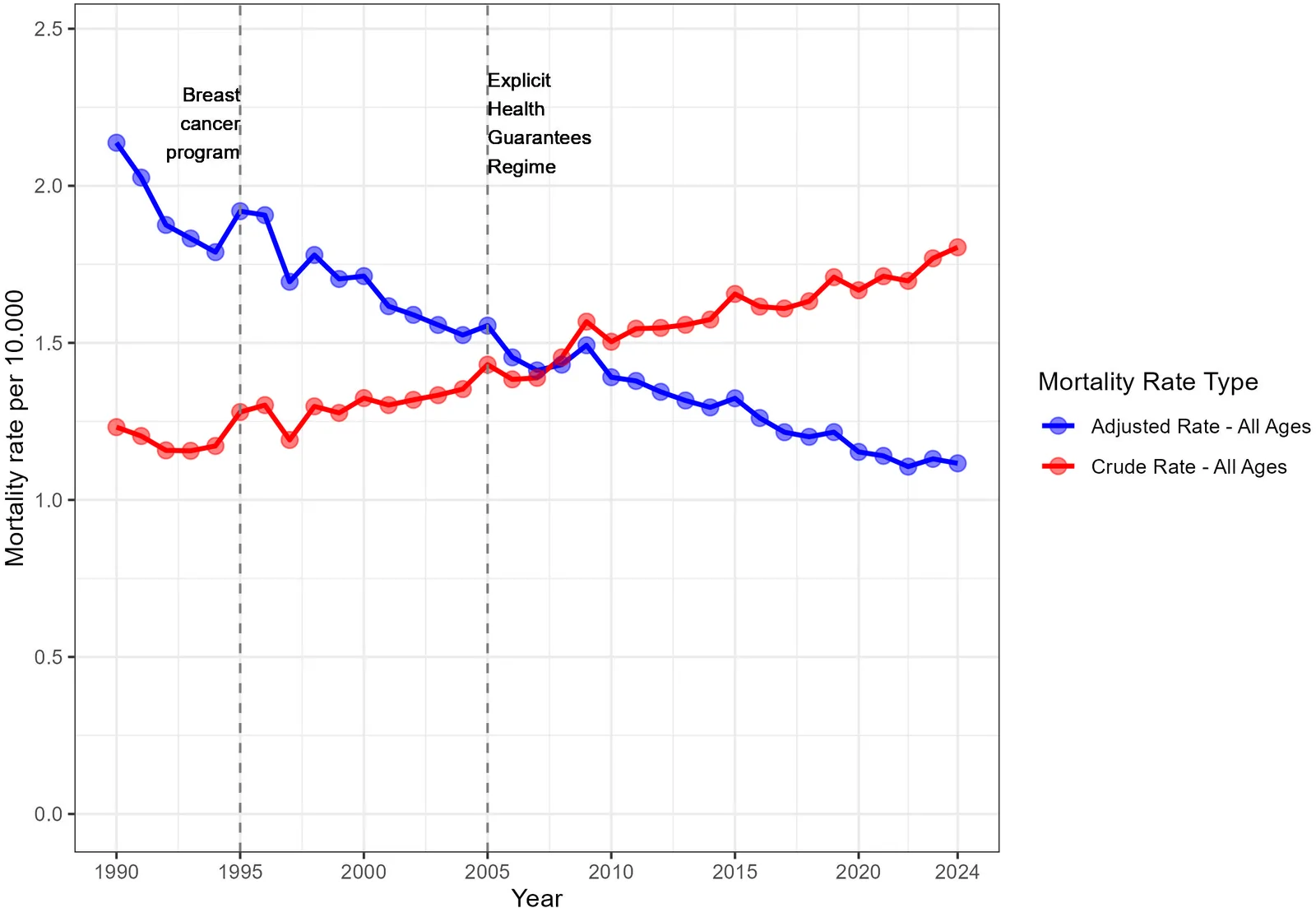
Crude and age-adjusted breast cancer mortality rate in female population, Chile 1990-2024.

Following the Breast Cancer Program in 1995, the age-adjusted mortality rate showed a sustained decline after a brief period of increasing rates (1995-1996). After the implementation of AUGE/GES in 2005, the adjusted mortality appears to have a sustained declining trend, likely through improved access to standardized diagnostic and therapeutic protocols.

The 80+ age group represents the single highest burden of mortality, accounting for 22% of all female deaths (n = 9,510). This group maintained the highest mortality throughout the study period, with the maximum rate of 15.8 per 10,000 in 1996 and a minimum rate of 10.75 in 2017. While rates for under 50 remain negligible, a significantly increased rate is observed starting at age 50, with an age-mortality gradient. The groups between ages 50 and 79 collectively represent 60% of the total mortality burden. After 1995 (Breast Cancer Program) and 2005 (AUGE/GES), the mortality rate in the 80+ and 65–69 age groups declined. The other age groups did not show a clear pattern (**Figures 2**, **3**).

**Figure 2:**
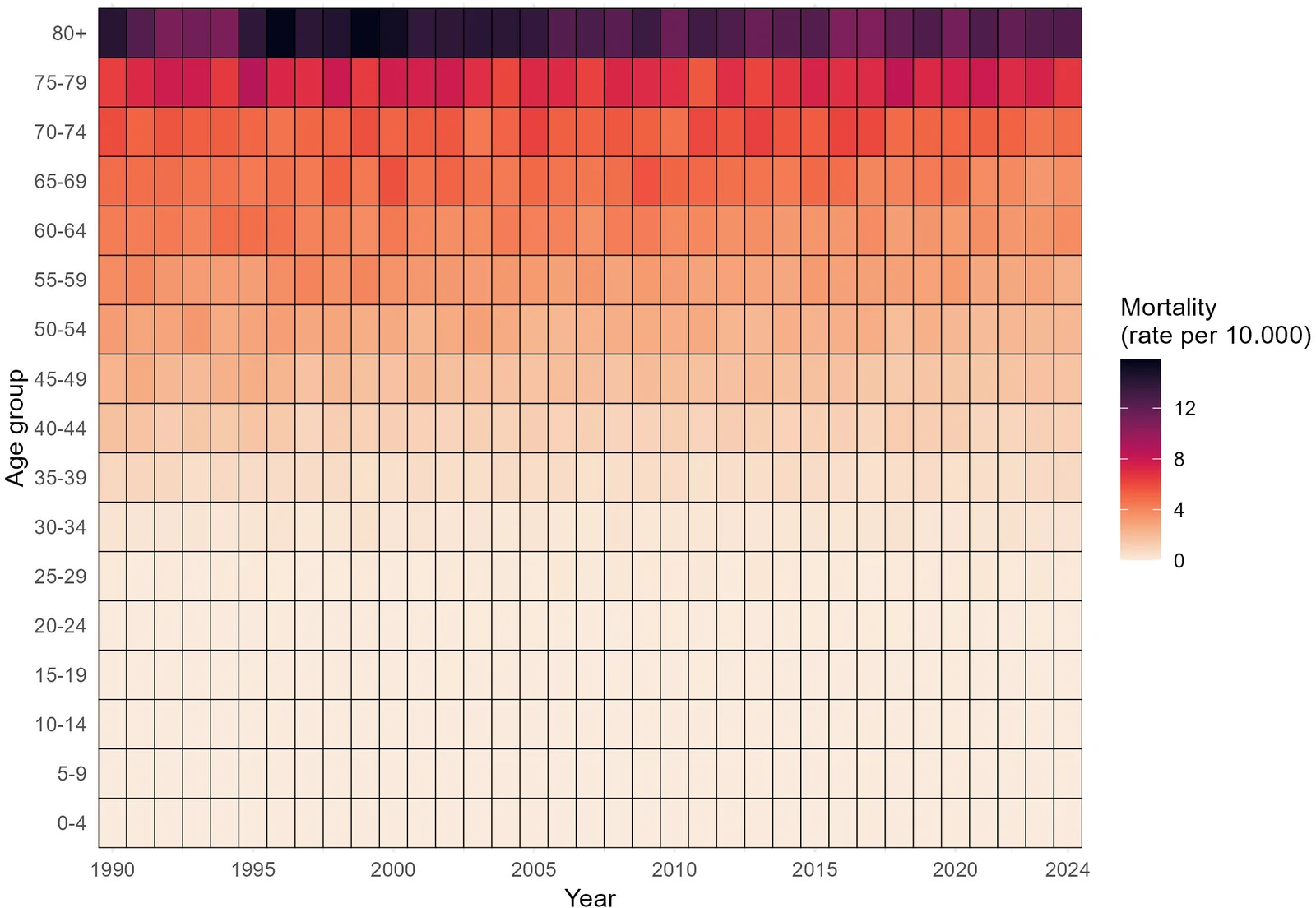
Age-specific breast cancer mortality rate in female population, Chile 1990-2024.

**Figure 3:**
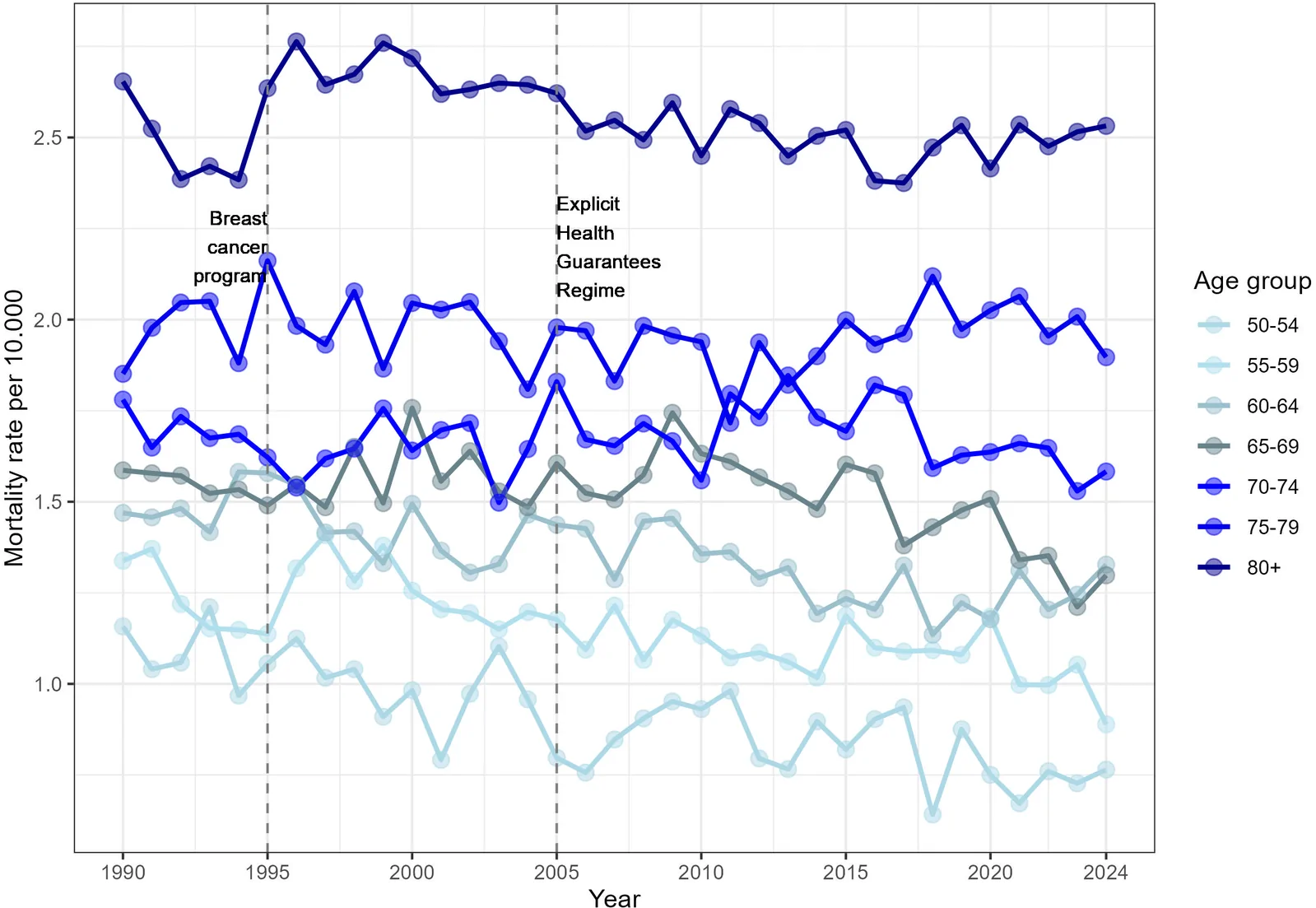
Age-specific breast cancer mortality rate in 50+ female population, Chile 1990-2024.

To assess the impact of the 1995 Breast Cancer Program and the AUGE/GES on mortality trends for the total female population, two interrupted time series were performed, one for each intervention. We did not find severe overdispersion in any of the models, a moderate overdispersion was found on the model for women ≥80 and only one model (women 65-69) showed autocorrelation (p-value = 0.049)(**Supplementary Material**).

The first model to assess the Breast Cancer Program showed that the mortality rate was significantly decreasing before 1995 with an RR of 0.955/year. An immediate non-significant change in the mortality rate was found in 1995 with the implementation of the breast cancer program (RR = 1.10, CI 95% 1.03 - 1.16). The absolute post-intervention trend declined with a significant RR of 0.981/year after the intervention (exp (β1 = -0.0459 + β3 = 0.0277)) at a slower rate than the pre-existing trend (**Table 2a**).

**Table 2:** Interrupted time series regression coefficients for age-standardized breast cancer mortality in the total female population of Chile, 1990–2024.

a. Model 1 breast cancer program: intervention starting in 1995				
Variable	Coefficient	Estimate	Rate Ratio (CI95%)	p-value
Pre-intervention (trend 1990–2004)	β1	-0.0459	0.955 (CI95% 0.971 - 0.939)	<0.001**
Breast Cancer Program (Immediate change in level 1995)	β2	0.0917	1.10 (CI95% 1.05 - 1.03)	0.0029*
Post-intervention trend	β3	0.0277	1.03 (CI95% 1.05 - 1.01)	0.0014*
b. Model 2 Explicit Health Guarantees (AUGE/GES) regime in 2005: intervention starting in 2005				
Variable	Coefficient	Estimate	Rate Ratio (CI95%)	p-value
Pre-intervention (trend 1990–2004)	β1	-0.024	0.980 (CI95% 0.983 - 0.977)	<0.001**
AUGE**/**GES Regime Start (Immediate change in level 2005)	β2	0.010	1.01 (CI95% 1.05 - 0.972)	0.604
Post-intervention trend	β3	0.003	1.00 (CI95% 1.01 - 0.999)	0.101

*significant p-value < 0.05.**significant p-value < 0.01.

The second model, to assess the AUGE/GES, showed that the mortality rate was significantly decreasing before 2005 with a RR of 0.980/year (CI95% 0.983 - 0.977). The implementation of the AUGE/GES was associated with a non-significant change with a RR 1.01. Likewise, the post-intervention (β3) showed a non-significant trend with an RR of 1.00/year compared to the pre-existing trend. The post-intervention absolute trend decreased with a RR of 0.982/year after the intervention (exp (β1 = -0.0204 + β3 = 0.003), p-value < 0.05) at a slower rate than the pre-existing trend (**Table 2b**).

Analyses restricted to women aged 50 years and older yielded patterns similar to those observed in the total population. A change in trend was detected after 1995, followed by continued reductions in age-standardized mortality. As in the overall analysis, no significant change in level or slope was identified after 2005, indicating that mortality continued to decline along the pre-existing trajectory (**Tables 3a, b**).

**Table 3:** Interrupted time series regression coefficients for age-standardized breast cancer mortality in women aged ≥50 years, Chile, 1990–2024.

a. Model 3 breast cancer program: intervention starting in 1995				
Variable	Coefficient	Estimate	Rate Ratio (CI95%)	p-value
Pre-intervention (trend 1990–2004)	β1	-0.055	0.946 (CI95% 0.966- 0.927)	<0.001**
Breast Cancer Program (Immediate change in level 1995)	β2	0.005	1.06 (CI95% 1.14 - 0.927)	<0.001**
Post-intervention trend	β3	0.022	1.02 (CI95% 1.04 - 1.00)	0.0379 *
b. Model 4 Explicit Health Guarantees (AUGE/GES) regime in 2005: intervention starting in 2005				
Variable	Coefficient	Estimate	Rate Ratio (CI95%)	p-value
Pre-intervention (trend 1990–2004)	β1	-0.037	0.963 (CI95% 0.966 - 0.960)	<0.001**
AUGEGES Regime Start (Immediate change in level 2005)	β2	-0.019	0.981 (CI95% 1.02 - 0.943)	<0.001**
Post-intervention trend	β3	0.008	1.01 (CI95% 1.01 - 1.01)	0.341

*significant p-value < 0.05. **significant p-value < 0.01.

We fitted two models for each of the seven age groups of women over 50 years old. Our results indicate that the Breast Cancer Program did not significantly reduce the mortality rate trend in any of these groups. However, following the implementation of AUGE/GES, Interrupted Time Series (ITS) models identified a significant change in the trend for two specific groups compared to their pre-intervention trajectories: women aged 65–69 experienced a 1.9% annual decline (RR = 0.981, 95% CI: 0.967–0.996; **Tables 4a, b**), and women aged 80 and older experienced a 1.5% annual decline (RR = 0.985, 95% CI: 0.974–0.996; **Tables 5a, b**). In absolute terms, the post-AUGE/GES period showed a significant annual mortality decline of 1.8% for women aged 65–69 (RR = 0.982, exp(β1 = 0.001 + β3 = -0.018)). For the 80 and older group, the absolute annual reduction was 0.32%, which was not statistically significant (RR = 0.996, exp(β1 = 0.012 + β3 = -0.015)).

**Table 4:** Interrupted time series regression coefficients for age-standardized breast cancer mortality for age group 65–69 years, Chile, 1990–2024.

a. Model 5 breast cancer program: intervention starting in 1995				
Variable	Coefficient	Estimate	Rate Ratio (CI95%)	p-value
Pre-intervention (trend 1990–2004)	β1	-0.016	0.984 (CI95% 1.060 - 0.913)	0.676
Breast Cancer Program (Immediate change in level 1995)	β2	0.144	1.16 (CI95% 1.50 - 0.897)	0.271
Post-intervention trend	β3	0.006	1.01 (CI95% 1.09- 0.934)	0.857
b. Model 6 Explicit Health Guarantees (AUGE/GES) regime in 2005: intervention starting in 2005				
Variable	Coefficient	Estimate	Rate Ratio (CI95%)	p-value
Pre-intervention (trend 1990–2004)	β1	0.001	1.00 (CI95% 1.01 - 0.988)	0.8564
AUGE/GES Regime Start (Immediate change in level 2005)	β2	0.093	1.10 (CI95% 1.27 - 0.954)	0.1935
Post-intervention trend	β3	-0.018	0.981 (CI95% 0.996 - 0.967)	0.0138*

* means a significant p value < 0.05.** means a significant p value < 0.01.

**Table 5:** Interrupted time series regression coefficients for age-standardized breast cancer mortality for age group 80+ years, Chile, 1990–2024.

a. Model 7 breast cancer program: intervention starting in 1995				
Variable	Coefficient	Estimate	Rate Ratio (CI95%)	p-value
Pre-intervention (trend 1990–2004)	β1	-0.065	0.937 (CI95% 0.996 - 0.882)	0.0356*
Breast Cancer Program (Immediate change in level 1995)	β2	0.398	1.49 (CI95% 1.84 - 1.21)	<0.001**
Post-intervention trend	β3	0.05703	1.06 (CI95% 1.13 - 0.996)	0.0664
b. Model 8 Explicit Health Guarantees (AUGE/GES) regime in 2005: intervention starting in 2005				
Variable	Coefficient	Estimate		p-value
Pre-intervention (trend 1990–2004)	β1	0.012	1.01 (CI95% 1.02 - 1.00)	0.01742*
AUGE/GES Regime Start (Immediate change in level 2005)	β2	-0.175	0.839 (CI95% 0.928 - 0.758)	<0.001**
Post-intervention trend	β3	-0.015	0.985 (CI95% 0.974 - 0.996)	0.006**

*significant p-value < 0.05.**significant p-value < 0.01.

In summary, the results indicate that Absolute breast cancer deaths and crude mortality rates increased over time, in contrast age-standardized mortality declined consistently from 1990 onward. A significant absolute and declining trend occurred after 1995, coinciding with earlier programmatic organization, and no additional population-level changes were observed after the 2005 implementation of AUGE/GES. Reductions in mortality after 2005 were more evident in older age groups (65–69 and ≥80 years), suggesting age-specific heterogeneity.

## Discussion

This interrupted time series (ITS) study used negative binomial regression to assess whether the 2005 incorporation of breast cancer into Chile’s Explicit Health Guarantees (AUGE/GES) was associated with a detectable change in national breast cancer mortality trends, by comparing post-intervention level and slope with the pre-existing trajectory (18, 21, 22). Overall, the findings indicate that age-standardized mortality was already declining before 2005 and that the introduction of AUGE/GES was not associated with an additional aggregate-level inflection in national trends. This pattern is consistent with policy interventions that consolidate access, financing, and clinical processes without producing an abrupt shift in mortality when strong secular declines are already underway (25–28).

Taken together, the results suggest that AUGE/GES primarily functioned as a mechanism of consolidation and protection, rather than as a driver of accelerated mortality reduction beyond pre-existing long-term trends. In this context, structural and social determinants, along with screening organization and coverage, remain central drivers of population-level outcomes.

A key interpretive finding is the divergence between crude and age-standardized indicators. While absolute numbers of deaths and crude mortality rates increased, age-standardized rates declined—an expected pattern in settings undergoing rapid demographic aging and epidemiologic transition (25–27, 29, 30). In Chile, population aging likely masks improvements in age-specific survival and clinical management, as a growing share of women enter older, higher-risk age groups (29, 30). This interpretation is consistent with national epidemiological analyses describing sustained declines in adjusted mortality risk alongside persistent challenges related to advanced-stage burden and health system constraints (3, 5, 27, 28).

### Timing of inflections: 1995 vs. 2005

The ITS analyses indicate that an absolute annual decline was present after 1995 and 2005, temporally aligned with the consolidation of the National Breast Cancer Program and the organization of preventive and diagnostic services and AUGE/GES services. After interventions mortality continued to decline, although at a slower pace, suggesting sustained gains along a trajectory that was already improving (2, 3, 9, 14, 25–27).

No population-level inflection was detected in 1995 or 2005 compared to previous years in aggregated national age-standardized trends (3, 5, 18, 21, 25–28). However, age-stratified analyses revealed heterogeneity: women aged 65–69 years and those aged ≥80 years experienced more pronounced declines after 2005, without a comparable change around 1995. This pattern is compatible with the hypothesis that treatment-related components of AUGE/GES yielded more visible benefits in older, higher-risk cohorts, while producing limited detectable incremental change in national aggregate indicators (3, 5, 25–28).This interpretation aligns with prior national accounts of programmatic strengthening in the 1990s and progressive clinical/therapeutic improvements in subsequent decades (2, 3, 5, 9, 14).

Although breast cancer mortality was concentrated among women aged ≥50 years, the burden among younger women should not be considered negligible. In this series, 7,552 deaths (17.4% of female deaths) occurred before age 50, including 1,983 deaths (4.6%) among women aged <40 years (**Table 1**). This subgroup is particularly relevant in Latin America, where young women account for a greater proportional burden of breast cancer incidence and mortality than in higher-income settings and are more frequently diagnosed with advanced-stage, high-grade, triple-negative, or HER2-positive tumors (31, 32). Young age has also been associated with poorer breast cancer-specific survival in a subtype-dependent manner, particularly among women with luminal tumors (33). Because the present ITS analysis did not model women aged <50 years as a separate stratum, the effect of AUGE/GES on younger women cannot be inferred from the reported age-specific estimates. Future studies should therefore examine this population separately and incorporate stage at diagnosis, molecular subtype, diagnostic delay, treatment completion, and long-term survival.

### Equity and continuum-of-care constraints

Persistent inequities across the continuum of care provide a plausible explanation for the attenuated population-level effect of AUGE/GES. National studies document substantial insurance-related gaps in outcomes, with approximately 90% five-year survival among women covered by private insurance (ISAPRE) compared with ~81% among those insured by the public system (FONASA), alongside marked differences in five-year case fatality (~16% vs ~27.5%, respectively) (34). Additional evidence indicates systematic differences in treatment timeliness by insurance type, suggesting that formal guarantees do not always translate into effective, timely, and high-quality care across the system (35).

Mammography use in Chile also exhibits persistent socioeconomic gradients, limiting the potential mortality impact in the absence of organized and equitable screening coverage (6). Qualitative and mixed-methods studies further identify structural and organizational barriers that impede timely diagnosis and treatment, reinforcing the importance of continuum-oriented interventions (7). At the regional level, breast cancer outcomes in Latin America reflect the combined influence of socioeconomic, clinical, and system-level determinants, underscoring the limitations of isolated policy guarantees (36).

Several non-mutually exclusive mechanisms may explain why a clear population-wide “AUGE/GES effect” on mortality is not observed. First, strong secular declines linked to earlier programmatic strengthening and progressive therapeutic advances may reduce the statistical detectability of incremental policy effects when the underlying trend is already improving (2–5, 25–28). Second, mortality is a distal and lagged endpoint that integrates multiple steps across the continuum of care; improvements in financial protection or access may not translate into measurable population mortality shifts without high and equitable coverage of early detection, timely diagnostic resolution, and effective treatment completion (18, 21, 37). Third, screening coverage remains incomplete and socially patterned, limiting the potential for large population-level mortality reductions observed in settings with fully organized screening programs and recall systems (6, 37–41). Fourth, persistent inequities in timeliness and capacity across a segmented public–private system may attenuate the translation of formal guarantees into uniform outcomes (7, 34, 35). Finally, benefits may concentrate in specific age groups, territories, or service networks with higher care capacity, leaving national aggregate indicators less sensitive to heterogeneous improvements (30, 34, 35).

International evidence suggests that the largest and most consistent declines in breast cancer mortality occur where health systems close the continuum of care through a combination of organized population-based screening (nominal invitation, recall, and quality assurance), fast-track diagnostic–treatment pathways with enforceable timelines, coordinated multidisciplinary oncology care, and broad access to effective therapies (37).

Global surveillance initiatives report substantial cross-country variation in breast cancer survival, with many high-income European settings reaching or exceeding ~85% 5-year survival, while lower survival is observed in parts of Latin America and other regions (42). OECD syntheses also highlight that mortality trajectories for several cancers (including breast cancer) can diverge across health systems depending on prevention, early detection, and treatment organization (17, 43).

International evidence suggests that mortality reductions are greatest when screening is organized at the population level and integrated with timely diagnostic resolution and effective treatment. Studies from European and Asian health systems have reported mortality benefits associated with organized mammography programs, although effect sizes vary according to population, design, screening uptake, and health-system organization (38–41, 44–51). These experiences reinforce the importance of evaluating screening not as an isolated intervention but as part of an integrated continuum of cancer care.

Compared with these settings, Latin American countries—including Chile—often operate with more fragmented screening and persistent bottlenecks across diagnostic and treatment pathways, which may contribute to later-stage diagnosis and poorer outcomes.

The Chilean experience captured here is consistent with a model in which early program organization (mid-1990s) contributed to the primary shift in national mortality risk, while AUGE/GES functioned mainly as a consolidation mechanism with age-heterogeneous benefits that are partially masked by secular trends and persistent inequities.

### Strengths and limitations

This study leveraged a long national time series and applied ITS methods that explicitly account for pre-intervention trends, using negative binomial regression appropriate for over-dispersed mortality counts (18, 21, 22). Several limitations should be noted. First, the ecological design precludes individual-level causal inference and does not allow direct evaluation of clinical mediators such as stage at diagnosis, tumor subtype, treatment adherence, or completion. Second, mortality does not distinguish between changes in incidence, survival, or case fatality and may be influenced by concurrent improvements in therapy and potential changes in certification or registration practices. Third, AUGE/GES was implemented alongside other contemporaneous changes (technology diffusion, clinical guidelines, oncology capacity), and therefore the estimated association represents a net, aggregated effect (3, 5, 25, 28). Finally, screening coverage and diagnostic–treatment timeliness were not directly modeled; incorporating time series on mammography uptake, stage distribution, and waiting-time indicators could clarify the mechanisms linking policy to outcomes (6, 7, 34, 35, 51).

### Future research

Future research should extend these analyses by: (i) stratifying ITS models by territory and insurance type to quantify heterogeneity and equity gaps more directly (34, 35); (ii) integrating intermediate indicators across the continuum of care (stage at diagnosis, time to biopsy, time to treatment initiation, treatment completion), complementing mortality as a distal endpoint (6, 37, 51); and (iii) conducting equity-focused evaluations that incorporate socioeconomic determinants and effective access to oncology networks, linking policy implementation to measurable changes in timely, high-quality care (6, 7, 34, 35, 51).

## Conclusions

Breast cancer mortality risk in Chile declined over the last three decades, while absolute deaths and crude mortality increased, consistent with demographic aging (25–27, 29, 30). The main inflection in national mortality trends aligns temporally with programmatic strengthening in the mid-1990s (2, 9, 14), and subsequent national evidence supports sustained improvements alongside persistent continuum challenges (3, 5, 28). The 2005 AUGE/GES reform has consolidated gains—potentially with age-specific benefits in older cohorts—without generating an additional detectable break in national age-standardized mortality trends at the aggregate level (3, 5, 18, 21, 25–28). Further reductions are likely to require integrated, equity-oriented strategies that strengthen prevention, organized screening, timely diagnosis, and high-quality treatment across the life course, particularly given persistent insurance and socioeconomic gaps in outcomes and access (6, 7, 34, 35, 37).

## Policy implications: from “guarantees” to “outcomes”

To achieve additional mortality reductions, Chile may need to complement access guarantees and financial protection with a results- and equity-oriented package that strengthens the full care continuum (37):

- Organized population-based screening with nominal invitations, recall systems, quality assurance, and monitoring of effective coverage, with targeted outreach for groups facing lower access and persistent socioeconomic gradients (6).
- Fast-track diagnostic and treatment pathways with enforceable timelines, traceability, and coordination across levels of care, supported by multidisciplinary teams, consistent with pathway-based models that reduce delays and improve patient experience (44–46).
- Reduction of public–private gaps in waiting times, stage at diagnosis, and access to high-value therapies, given persistent disparities in survival and case fatality by insurance (7, 34, 35).
- Routine measurement of continuum indicators (time to diagnostic resolution, time to treatment initiation, stage distribution, adherence and completion), complementing mortality as a late endpoint (6, 7, 34, 35, 37, 51).

Recent reforms, including Law 21.551, which removes the requirement for a physician’s order for screening mammography and establishes a provider duty to ensure access, may reduce barriers to care. However, their impact will depend on service capacity, territorial organization, patient navigation, and the timeliness and effectiveness of downstream diagnostic and treatment pathways (52). Because increases in absolute deaths partly reflect population aging and longer life expectancy, policy should also strengthen primary prevention and explicitly expand geriatric oncology capacity to meet the needs of older adults (29, 30, 37).

## Data Availability

The datasets presented in this study can be found in online repositories. The names of the repository/repositories and accession number(s) can be found in the article/**Supplementary Material**.
